# Evidence for Independent Processing of Shape by Vision and Touch

**DOI:** 10.1523/ENEURO.0502-21.2022

**Published:** 2022-06-14

**Authors:** Ryan L. Miller, David L. Sheinberg

**Affiliations:** 1 Department of Neuroscience, Brown University, Providence, Rhode Island 02912; 2 Carney Institute for Brain Science, Brown University, Providence, Rhode Island 02912

**Keywords:** cross-modal, haptic, multisensory, object recognition, supramodal, visual

## Abstract

Although visual object recognition is well studied and relatively well understood, much less is known about how shapes are recognized by touch and how such haptic stimuli might be compared with visual shapes. One might expect that the processes of visual and haptic object recognition engage similar brain structures given the advantages of avoiding redundant brain circuitry and indeed there is some evidence that this is the case. A potentially fruitful approach to understanding the differences in how shapes might be neurally represented is to find an algorithmic method of comparing shapes, which agrees with human behavior and determines whether that method differs between different modality conditions. If not, it would provide further evidence for a shared representation of shape. We recruited human participants to perform a one-back same–different visual and haptic shape comparison task both within (i.e., comparing two visual shapes or two haptic shapes) and across (i.e., comparing visual with haptic shapes) modalities. We then used various shape metrics to predict performance based on the shape, orientation, and modality of the two stimuli that were being compared on each trial. We found that the metrics that best predict shape comparison behavior heavily depended on the modality of the two shapes, suggesting differences in which features are used for comparing shapes depending on modality and that object recognition is not necessarily performed in a single, modality-agnostic region.

## Significance Statement

Humans are adept at recognizing objects by touch alone despite the inherent complexity required to integrate information from touch receptors across multiple articulating fingers. Little is understood about how this is accomplished and to what extent the brain borrows visual object recognition machinery to achieve this goal. Here we use various metrics for predicting human shape comparison behavior and find that the best metrics vary considerably depending on the modality (vision or touch) used to evaluate the shapes. This suggests that there may be more independence between unfamiliar visual and haptic object recognition processing than previously believed.

## Introduction

Object recognition is a core capacity afforded by our visual system and, accordingly, has long been of great interest to psychologists, neuroscientists, and philosophers (Cheselden, 1728; Ettlinger, 1956; Bülthoff et al., 1995; Riesenhuber and Poggio, 1999; Martin, 2007; Peissig and Tarr, 2007; Marr, 2010). The ability to recognize objects is not exclusively a visual faculty, however; we are also quite adept at recognizing objects solely by touch when, for example, searching for a coin in a pocket or purse. Although we know a great deal about the role that somatosensory pathways play in the perception of basic dimensions of touch, such as texture and vibration (Klatzky et al., 1985; Lederman and Klatzky, 1987; Sathian, 2016), little is understood about how the brain ultimately integrates this information to serve advanced functions such as haptic object recognition.

From one perspective, both visual and haptic object recognition might be assumed to be processed by shared neural circuitry. After all, object recognition taps the same basic ability, regardless of the source modality, and it would seem economical not to have duplicate machinery. Indeed, this view has received support from groups studying the human visual extrastriate regions such as lateral occipital cortex and inferotemporal cortex using imaging (Grill-Spector et al., 1998; Amedi et al., 2001; James et al., 2002; Pietrini et al., 2004; Prather et al., 2004; James and James, 2005; Lee Masson et al., 2016; but see Snow et al., 2015).

Alternatively, it is clear that visual and somatosensory signals originate from fundamentally different end organs and are, at least initially, processed independently. So, from this perspective, recognition of objects using information from these senses may be contained within their own modality-specific circuits. Further complicating the issue, we know it is possible to compare shapes that are perceived haptically with shapes perceived visually, meaning that there must be some way for the neural machinery processing these unisensory stimuli to communicate shape information. If visual and haptic shapes are processed in the same areas, this comparison may be relatively simple. If processed separately, comparisons may only be possible through intermediaries such as classical association or prefrontal areas responsible for higher-level cognition. Researchers investigating anterior intraparietal cortex and dorsolateral prefrontal cortex have found evidence to support this, finding these areas to be especially active when comparing shapes across modalities (Murata et al., 2000; Grefkes et al., 2002; Ricciardi et al., 2006; Lacey et al., 2010; Helbig et al., 2012).

Yet another possibility is something of a middle ground: that the extent of visual cortical involvement in haptic shape recognition is dependent on other factors such as familiarity. The importance of familiarity in haptic processing has been highlighted by a number of behavioral studies (Ikeda and Uchikawa, 1978; Magee and Kennedy, 1980; Lederman et al., 1990; Ballesteros et al., 1999) and neuroimaging studies (Deshpande et al., 2010; Lacey et al., 2010; Cacciamani and Likova, 2016).

**Figure 1.**
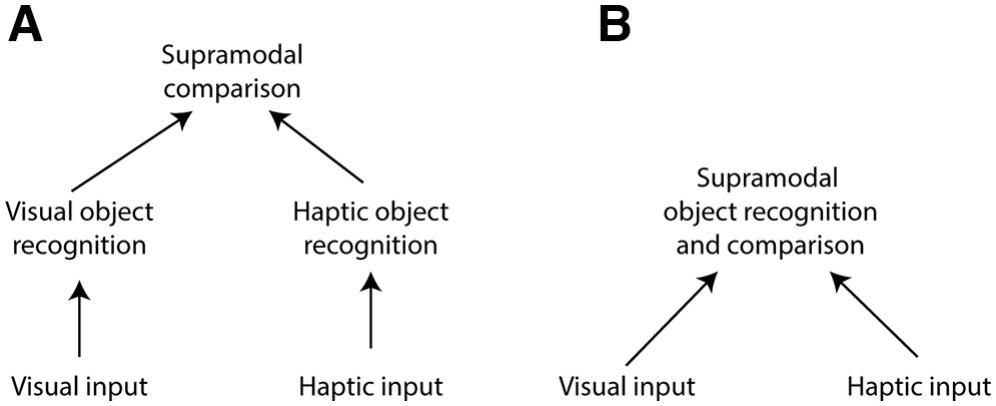
Schematized illustration of alternative hypotheses. ***A***, Late-convergence model. One possibility is that shapes are recognized independently within unisensory processing areas and, if necessary, compared in some higher “supramodal” processing area. ***B***, Early-convergence model. Alternatively, object recognition for the two modalities may use shared neural machinery following basic feature extraction (e.g., lines and curvature) in unisensory areas. Adapted from the study by Lacey et al. (2009).

Here we attempt to disentangle these possibilities by determining the extent to which shapes from the two modalities are similarly represented. Figure 1 illustrates two alternative hypotheses that we are seeking to differentiate. Following basic feature extraction in visual and somatosensory unisensory areas, little is known about how haptic object recognition is completed and how much of the related neural machinery overlaps with what are considered visual processing areas. To the extent that there is a great deal of overlap (“early-convergence model”), we might expect that the same shape features determined to be crucial for visual object recognition would also be crucial for haptic object recognition. On the other hand, if the properties that are relevant for recognizing visual shapes are much different from those for haptic shapes, we might conclude that the neural underpinnings of these two abilities are substantially different (“late-convergence model”). Furthermore, mistakes in object recognition can be informative. If there is a distinct difference in the types of mistakes made when identifying shapes using the two different modalities, this would be evidence that the pathways responsible may be substantially different.

We recruited human participants to perform a one-back shape-matching task that involved determining whether a given two-dimensional (2D) abstract shape is the same as or different from the shape that was presented seconds earlier. This was done both within modalities (i.e., comparing visual with visual, haptic with haptic) and across modalities (i.e., comparing visual and haptic shapes). This necessitated the design and production of a new device capable of quickly and reliably presenting physical objects from a large inventory (see Materials and Methods).

## Materials and Methods

Human participants (*n* = 10, 8 female) were recruited from Brown University undergraduate and graduate student populations to perform a visual–haptic matching task lasting approximately 1 h and were paid $15. Methods were approved by the Brown University Institutional Review Board. All participants had normal or corrected-to-normal vision. All participants were right handed. One participant was excluded from this study because of miscommunication of instructions.

### Task

Participants performed a one-back task where they were asked to determine whether the current stimulus was the same shape as the previous stimulus and report their answer by pressing the button corresponding to “same” or “different.” Stimuli were presented one at a time in blocks of 72 trials. Participants sat with their heads resting in a chin rest for all conditions, and all trials began with a fixation point appearing until fixation was acquired, then the fixation point disappeared and either a visual stimulus was presented or they were free to touch the haptic stimulus. Orientation of the visual or haptic shape was pseudorandomly chosen on each trial so that there was a 1:1:1 ratio between matching trials that were the same orientation, trials that were rotated 90° left or right, and trials which were rotated 180°. Each trial was pseudorandomly chosen as same or different so that each block of 72 trials used 48 unique shapes and had 24 same trials. Within those constraints, there was no limit on consecutive same or different trials. For example, it was possible (though exceedingly unlikely) to have the same shape presented five times in a row. Consistent with previous experiments comparing visual and haptic stimuli (Newell et al., 2001; Lacey et al., 2007, 2009; Tabrik et al., 2021), participants were given double the time to explore haptic (6 s) as visual (3 s), after which point the stimuli were removed. They could report their decision at any time during or after the stimulus presentation.

**Figure 2.**
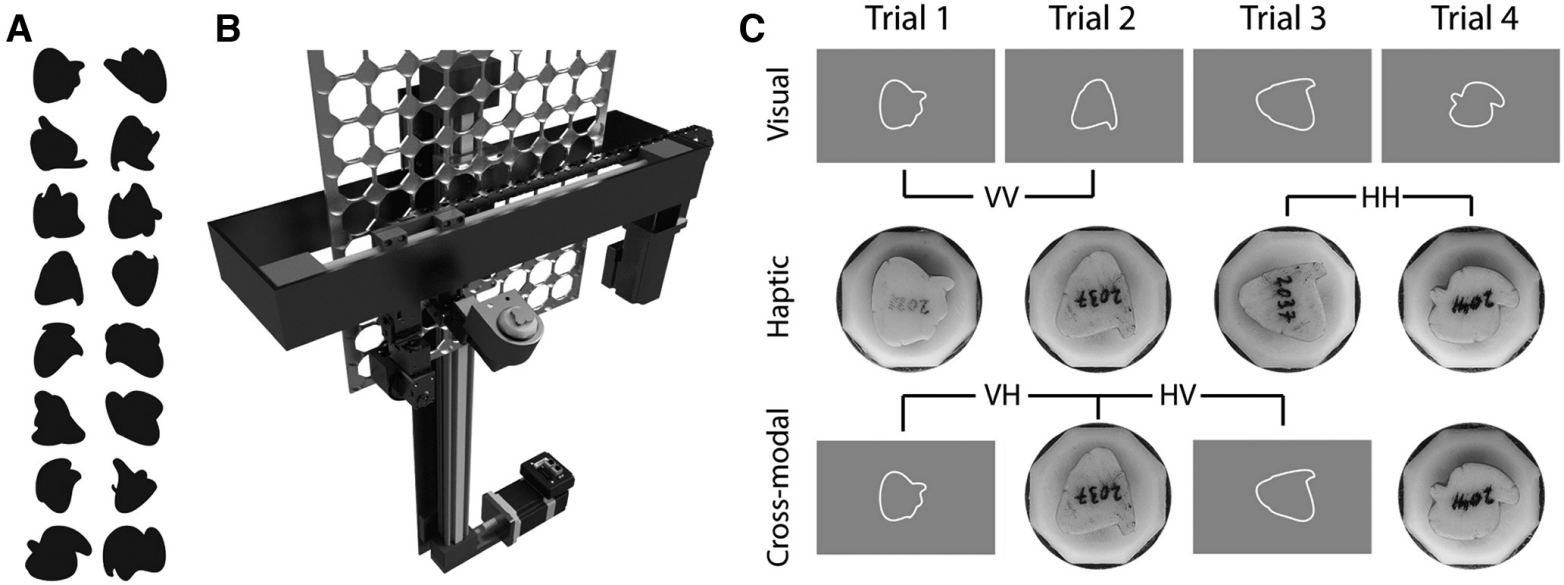
Stimuli and task. ***A***, Randomly selected 16 of the 48 shapes used in this task. ***B***, Rendering of the device used to present physical shapes to participants for haptic trials. ***C***, Task conditions. Each participant performed the one-back matching task in the following three block types: visual-only (top), haptic-only (middle), and cross-modal (bottom). These three block types yield the following four conditions: VV, HH, VH, and HV. Block types were completed in pseudorandom order, counterbalanced across participants. Each block consisted of 72 trials.

Each block was one of three types: visual-only, haptic-only, or alternating. “Alternating” blocks alternated between visual and haptic stimuli (Fig. 2*C*). These three block types provided four conditions, based on the within-modal or cross-modal comparison being made: the within-modal visual comparison (VV) trial and the within-modal haptic comparison (HH) trial. For cross-modal visual–haptic comparison (VH) trial, the visual shape presented on the previous trial is compared with the haptic shape on the current trial. For cross-modal haptic–visual comparison (HV) trials, the haptic shape presented on the previous trial is compared with the visual shape on the current trial. Each participant completed two visual-only blocks, two haptic-only blocks, and four alternating blocks, yielding 144 trials of each of the four conditions. The order of these eight blocks was counterbalanced across participants so as to avoid any order effect. Two-thirds of trials were different while the remaining one-third were same. Participants were given ∼20 practice trials for each of the three block types before beginning data collection.

### Visual stimuli

On each visual trial, an abstract white-outlined shape, centered at fixation, was presented on a uniform gray background. Each shape was scaled such that it was the same size (∼3° visual angle) as the haptic representation of that same shape and rotated to one of four orientations, spaced in 90° increments. The shapes were constructed in a manner similar to Sigurdardottir and Sheinberg (2015). Two or three two-dimensional “blobs” were generated using three randomly chosen control points connected by splines. Those blobs were then overlaid on each other and filled to produce a unique shape composed of multiple rounded edges (from the splines) and sharp corners (where two blobs intersect). Only unions of blobs that yielded a single filled shape were allowed. Examples of shapes used in the experiment are shown in Figure 2. Stimuli were presented on an LCD monitor (Display++, Cambridge Research Systems) with a 100 Hz refresh rate and eye position was tracked using an EyeLink eye tracker operating at 1 kHz to ensure fixation at the start of each trial.

### Haptic stimuli

A custom apparatus was designed and built (Fig. 2*B*) to hold an inventory of up to 80 unique objects and present any 1 or 2 of those 80 objects at a given time (only 1 object was presented at a time in the present experiment) to either the left or right hand. For this study, subjects explored the haptic shapes with their left hand, using their right hand to press one of two buttons indicating same or different. The haptic stimuli were positioned such that they could not be seen by the participant. The haptic presentation system includes an *x*–*y* slide system (drylin linear actuators, Igus) driven by stepper motors (Applied Motion) used to position the inventory panel and two arms each composed of three servo motors (DYNAMIXEL, ROBOTIS) used to retrieve, present, and return objects.

Each object could be independently rotated in plane for presentation at any angle. Each object was wrapped in 6 mm conductive foil tape (Adafruit), and that tape was divided into six sections, which were monitored at 100 Hz using a 12-channel capacitive touch sensor (6 channels available for each of two objects; model MPR121, Adafruit), enabling us to know when and where a shape was being touched by the participant.

On each haptic trial, a physical stimulus was presented to the left hand of participants at a comfortable position where their hand would naturally rest with elbows on their chair’s armrest, rotated to one of four 90° positions. Haptic stimuli were simply an extruded version of the two-dimensional visual stimulus. Each 2D shape was first scaled such that the maximum extent was 25 mm, then extruded 5 mm in depth using CAD (computer-aided design) software (Autodesk Fusion 360) and three-dimensionally (3D) printed (Mojo 3D printer, Stratasys). After 3D printing, the perimeter of the shape was wrapped in foil tape and divided into six sections, as described above. Each of those six touchpads was wired to a custom circuit board embedded within each object to make those touchpads electronically accessible. The entire object was then painted with conformal coating (MG Chemicals) to give a smooth, uniform feel and to protect the copper touchpads.

For this experiment, we specifically chose to present two-dimensional shapes, recognizing that these are only a subset of the kinds of objects encountered in the real world. For the visual–haptic comparisons under study, a significant advantage of using extruded 2D (as opposed to 3D) stimuli is that all relevant shape information is available to both modalities from a single view. With complex 3D shapes, a single view cannot reveal the entire shape (because you cannot see the back of an object) but a participants’ fingers would have access to that shape information, leading to a fundamentally different opportunity to perceive the shape.

When presenting physical stimuli (as opposed to digital stimuli rendered on a computer screen), care must be taken to avoid any possibility that the participant might gain additional helpful information from the sights or sounds generated by the presentation mechanism. For example, in the present same–different task, it would be trivially simple to perform perfectly just by listening to whether the machine picks up a new object (different trials) or not (same trials). Multiple steps were taken to address such confounds. First, the presentation device was obscured from view, thus providing no helpful visual information. Second, after each trial, when an object was returned to the panel holding the inventory of objects, it was returned to a new location. This prevented a participant from being able to guess the identity of an object by listening to the *x*–*y* travel of the machine. Third, on every haptic trial, whether it was a same trial (the same haptic stimulus needs to be presented on successive trials) or a different trial, an object was always dropped off and a different object was picked up. The only difference was that one robotic arm was used to drop off and pick up a new object on different trials and the second robotic arm was used to drop off and pick up a new sham object on same trials. The sham object was not actually presented to the participant, but there was no visual or auditory cue available to tell whether the real or sham object was presented, and thus no cue predicting whether the current stimulus was the same or different from the previous stimulus was present. Postexperiment questionnaires confirmed that participants had not found any strategy that was helpful in predicting the identity of haptic stimuli.

### Behavioral measurements and analysis

#### Shape measurements

One of the major goals of this study was to determine whether participants were more likely to mistake two different shapes as being the same shape if the two shapes were similar. The question then becomes: how do you define “similar”? Here, we selected a variety of metrics with which we can quantitatively evaluate and compare shapes that are intended to cover a wide range of plausible methods of comparison. We acknowledge that, although we have used a wide range of these metrics, our collection does not constitute an exhaustive list of possible metrics.

##### Distribution of angles

Each shape was defined by approximately 700–800 (depending on the perimeter length of each shape) points spaced at 0.1 mm increments. To calculate the distribution of angles for a given shape, the local angle at each of those points was calculated over a specified span. For example, for a span of 101 points, the angle at point *p* is calculated as the angle formed by the vectors from *p* to *p* – 50 and from *p* to *p* + 50. The distribution of all angles making up a shape is simply the cumulative density function (CDF) composed of these angles.

##### Aspect ratio

For a given shape, the *x* span (*x*max – *x*min) and *y* span (*y*max – *y*min) were calculated. Then, the aspect ratio at that rotation is calculated as *x*span/*y*span. The shape is then rotated at 1° increments, and the aspect ratio is computed again at each orientation. The aspect ratio for the shape is determined to be the largest of the 360 aspect ratios calculated for each shape.

##### Area/convex hull area/compactness

Area was calculated using the built-in MATLAB function “polyarea.” The convex hull was determined using the built-in MATLAB function “convhull,” and then the convex hull area was calculated using the function “polyarea.” Compactness is defined here as the area divided by the convex hull area.

#### Shape comparisons

##### Distribution of angles

To determine the similarity of two shapes, A and B, using this method, we computed the sum squared error between the CDFs of angles of shapes A and B.

##### Area/convex hull area/compactness

As with aspect ratio, we defined the similarity of two shapes in these measures to be the difference squared of the relevant measure.

##### Turning distance

This was calculated using the built-in MATLAB function “turningdist” based on the study by Arkin et al. (1991). Briefly, turning functions are calculated for each shape as the angle of the counterclockwise tangent as a function of the length of each segment of a shape, which is then normalized to a common length. This yields a complete representation of a shape that has the advantage of being invariant to size and *x*–*y* translation, but the disadvantage (for our application) of not being rotation invariant because the starting position for each turning function is arbitrary. Thus, the distance between turning functions is calculated for all starting positions of one shape and the minimum (min) distance is taken as the turning distance between two shapes.

##### Intersection over union

This was calculated using the built-in MATLAB functions “intersect” and “union,” with the area of intersection then divided by the area of union. Possible values range from 0 to 1, with 1 representing perfect overlap between shapes. This ratio is computed for the pair of shapes both for the actual orientations as presented (“@actual”) as well as at the optimal orientation that maximizes the overlap (“@optimal”) to represent the mental rotation a participant may be performing to attempt to align two shapes.

##### Aspect ratio

The similarity of the aspect ratios of two shapes is defined as the difference squared between the individual aspect ratios of the two shapes (@optimal method). The difference in aspect ratio is also measured under the assumption that no attempt at mental rotation is made (@actual), calculated as the aspect ratio of a bounding box with the long axis oriented the same as that for the shape on the previous trial. For example, if the shape presented on the previous trial had its long axis oriented 30° right of vertical, a participant may feel the current shape for a long axis that is ∼30° right of vertical.

##### Hausdorff distance

Shapes are first overlaid with aligned centerpoints. Next, for a given point on shape a, the nearest point on shape b was determined and the distance between these points was calculated. This was repeated for all points on each shape, and the maximum of these minimum distances is the Hausdorff distance. The Hausdorff distance is small for shapes that very nearly overlap and increase with larger deviations. Because this distance is not rotation invariant, it is calculated for both the actual orientations of the two shapes being compared (@actual) as well as the optimal orientation where the Hausdorff distance is minimized (@optimal) to account for the possible mental rotation a participant may perform to align two shapes.

### Metric evaluation

The various metrics were evaluated using a general linear model (GLM) with a binomial distribution to assess the relationship between the similarity of a pair of shapes (as determined by the metrics described above) with the response of a participant (same or different) on each trial. We used the Akaike information criterion (AIC) provided by the GLM as the dependent measure to evaluate a given metric. To facilitate comparisons within a condition (e.g., VH), a “random” metric was introduced that was simply a uniformly distributed random number assigned for each trial. For a metric to be considered predictive, it should provide at least an additional 3 units of AIC beyond the random metric (Burnham and Anderson, 2004). This use of AIC for assessment was particularly helpful when comparing the performance of metrics by themselves with models composed of multiple metrics, as it accounts for the likelihood that adding predictor variables will tend to improve model performance (purely by chance) by penalizing for the additional factors. Here we chose the model with the fewest variables that was not improved by at least 3 units by adding an additional variable.

### Monte Carlo simulation

To determine whether a given touchpad was touched more or less than would be expected by chance, and thus whether participants direct their haptic exploration toward particular features, we used a Monte Carlo simulation to form a baseline prediction of random touching. For each shape, 100,000 *x*–*y* points were randomly generated, each point representing a potential center point of a finger. If a given point was (1) outside the shape, (2) within 6 mm of an edge (representing the radius of a finger), and (*c*) not within 4 mm of an edge (representing the constraints on morphability of a finger when it touches a hard object), it was considered a touch. Any pad that was at least partially within 6 mm of the *x*–*y* point was considered “touched.” After 100,000 simulated touch points, the ratio of touches for each of the six touchpads was compared with the ratio of actual touches on those six touchpads to determine which pads were touched more or less than expected.

## Results

Ten participants participated in a one-back same–different task (Fig. 2) during which they evaluated a series of abstract shapes to determine whether each was the same as the last, regardless of changes in orientation. Each decision was signaled by pressing one of two buttons (same or different). Two-thirds of the trials were different trials, and the remaining one-third were same. Each participant completed 144 trials for each of the following four conditions: VV (comparing two visual shapes), VH (comparing a haptic shape on the current trial with a visual shape on the previous trial), HV (comparing a visual shape on the current trial with a haptic shape on the previous trial), and HH (comparing two haptic shapes).

### Analysis of within-modal and cross-modal conditions

**Figure 3.**
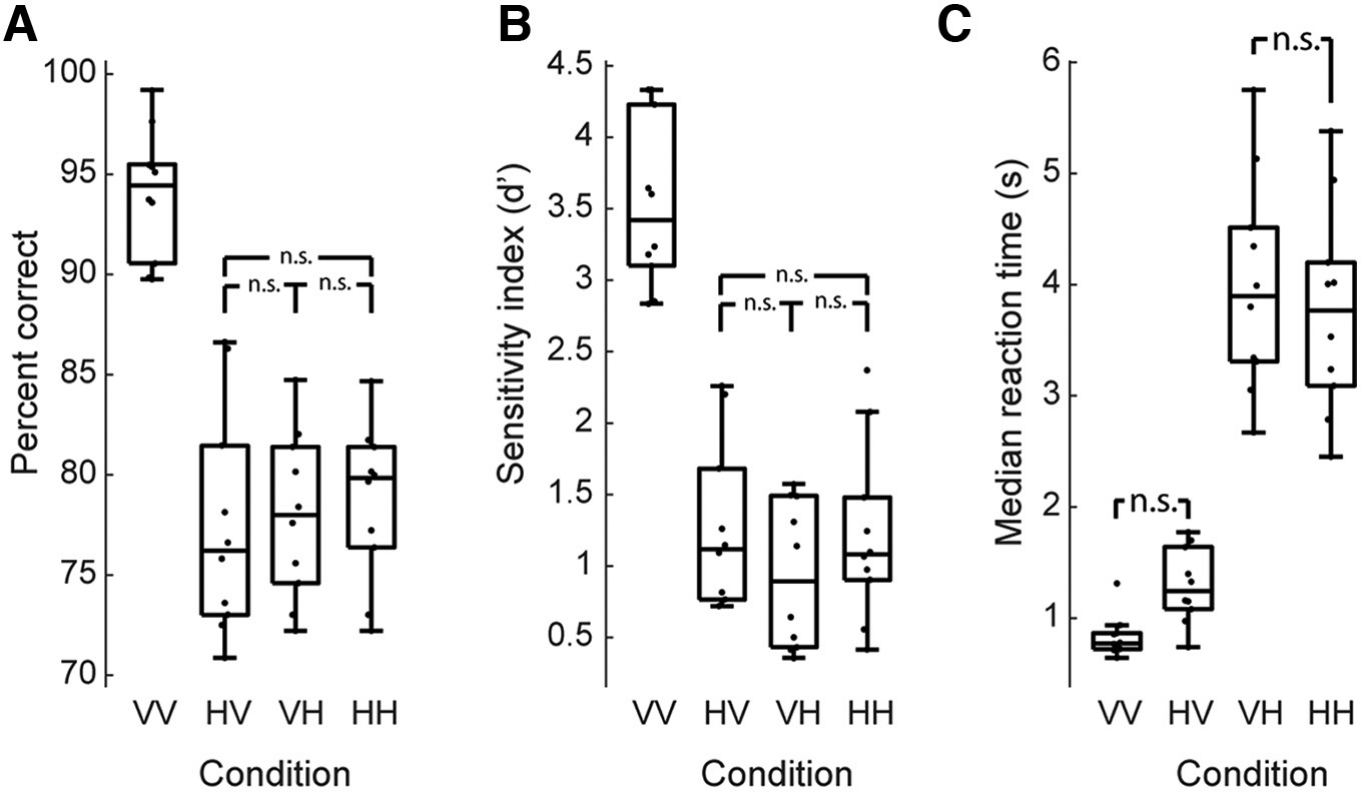
Task performance. All comparisons not labeled “n.s.” were significantly different (Tukey’s contrasts, *p* < 0.005). ***A***, Box plot showing performance (median, interquartile range, range) of all participants on each condition. Participants performed best on within-modal visual comparisons (VV). ***B***, Performance using *d*′ measure. ***C***, Median reaction time for each participant for each condition. Cross-modal responses tended to be slower than within-modal comparisons (*p* = 0.029), though each of the two cross-modal conditions (HV and VH) were not significantly slower than the comparable within-modal conditions (VV and HH, respectively).

We used a multilevel linear model to analyze the repeated-measures data. As is quite obvious from the results presented in Figure 3, the individual modalities used for the shape comparison (i.e., VV, HV, VH, and VV) had a significant effect on percentage correct performance (χ^2^ (3) = 55.84, *p* < 0.0001), sensitivity as measured using *d*′ (χ^2^ (3) = 61.84, *p* < 0.0001), and reaction time (χ^2^ (3) = 83.60, *p* < 0.0001). Performance in the within-modal conditions, compared with the cross-modal conditions, was better (for percentage correct: b = 4.30, *t*_(27)_ = 6.98, *p* < 0.0001; for *d*′: b = 0.637, *t*_(27)_ = 7.23, *p* < 0.0001), as would be expected from the high level of performance seen in the VV condition (Fig. 3). Indeed, using Tukey’s contrasts for multiple comparisons for performance and sensitivity, we also found that the VV condition was different from all three other conditions (all *z*-values less than −9.0, *p*-values < 0.0001), but that none of the other conditions differed significantly from each other.

Contrasts for the reaction time data provide a slightly different picture. Not surprisingly, as we found for performance and sensitivity, the modality of the current stimulus significantly affected reaction times, with responses to a currently presented visual stimulus being significantly faster than a current haptic stimulus (b = −1407, *t*_(27)_ = −18.82, *p* < 0.0001). However, the modality of the prior stimulus did not have a significant impact on reaction times (b = −58.5, *t*_(27)_ = −0.78, *p* = 0.44). We did find, though, that the within-modal conditions were overall faster than the cross-modal conditions (b = −172, *t*_(27)_ = −2.31, *p* = 0.029). We again used Tukey’s contrasts to compare the four conditions to each other, and we found that the VV condition differed from the VH and HH conditions (*z* values less than −14) but that although VV was slightly faster than the HV, the effect was not significant (estimate = 462, *z* = 2.3, *p* = 0.097). When comparing VH to HH, the within-modal haptic condition was faster (estimate = 228), but the difference was not significant (*z* = −1.135, *p* = 0.66). We note that the reaction time cost for comparing HV to VV is higher than comparing VH to HH. This suggests that translating a haptic representation in memory to compare to a visually presented match is more demanding than translating a visually stored shape into a haptic space.

### Effects of shape rotation

For match trials, the same shape was presented twice sequentially, but the orientation of that shape could differ on each of the two presentations. This was primarily to curtail certain undesirable strategies (e.g., feeling only the top left of each shape and comparing that small section between shapes), but it also allowed for an evaluation of the impact of rotation on recognition in different modalities. To assess the effects of rotation, and in particular to ask whether rotation had a different impact on the recognition of shape as a function of modality, we modeled the percentage correct and reaction time data using a factorial repeated-measures GLM (sensitivity could not be assessed as above as these were all match trials so we only have hits and misses). For this analysis, the first factor included levels for condition (VV, HV, VH, and HH). The second factor was stimulus rotation (0°, 90°, 180°). To test for the overall effect of each factor, we added them one at a time to the baseline model followed by inclusion of their interaction.

**Figure 4.**
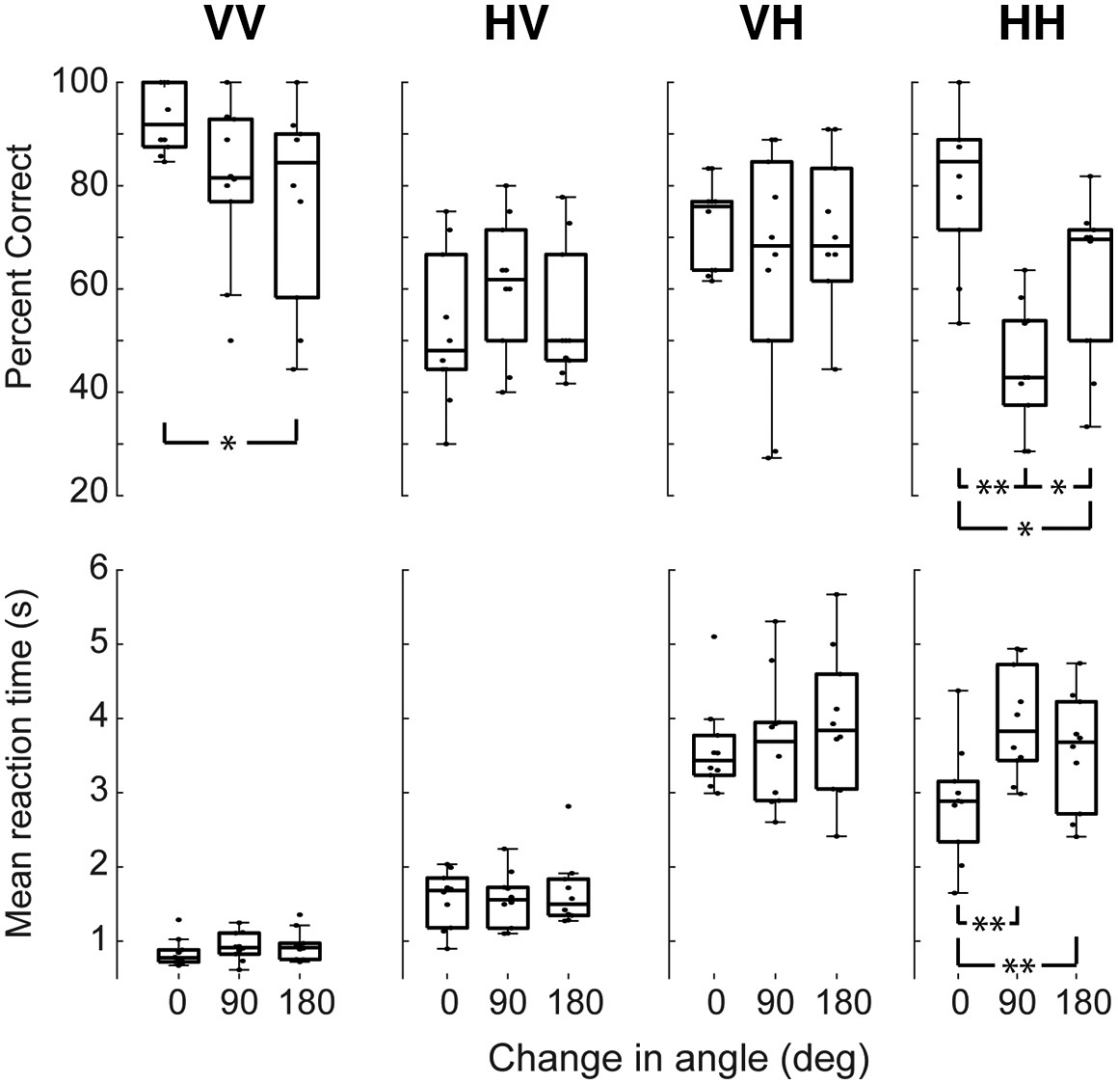
Performance with rotation of match shapes. Within-modal rotation tends to lead to more mistakes and longer reaction times, while no such penalty is seen for cross-modal comparisons. *p < 0.05; **p < 0.005; all others, *p* > 0.05.

Results for percentage correct performance are shown in Figure 4. Considering the hit rate, the addition of each factor and the interaction significantly improved the model fit (modality: χ^2^ (3) = 28,4, *p* < 0.001; rotation: χ^2^ (2) = 13.9, *p* = 0.0009; interaction: χ^2^ (6) = 30, *p* < 0.0001) and reaction time. This implies that performance across the four conditions differed, and that performance depended on the orientation of the sample and match stimulus. The significant interaction indicates that the effect of rotation itself depends on the modality.

Specific contrasts help us to better understand these trends. For the modality factor, we included orthogonal contrasts for “visual current” (VV and HV vs VH and HH), “cross-modal” (VV and HH vs HV and VH), and “unimodal pathway” (VV vs HH, ignoring the cross-modal conditions). For the rotation factor, we included contrasts for “rotated” (0° vs 90° and 180°) and for “degree of rotation” (90° vs 180°, ignoring 0°). All of these factors, except the last, were significant (visual current: b = −0.06, *t*_(27)_ = 2.72, *p* = 0.011; cross-modal: b = −0.05, *t*_(27)_ = 3.45, *p* < 0.005; within-modal pathway: b = 0.34, *t*_(27)_ = 5.34, *p* < 0.0001). These significant contrasts support the idea that, regardless of rotation difference, performance on match trials is better when the current stimulus is visual, when the comparison is made within modality compared with across modality, and, for unimodal trials (VV and HH), that visual comparisons are more accurate than haptic comparisons.

For the rotation contrasts, we found a significant effect of rotation away from 0° (rotated: b = −0.11, *t*_(27)_ = 4.41, *p* < 0.0001) but no overall difference between the 90° and 180° of rotation (degree of rotation: b = −0.02, *t*_(27)_ = 0.75, *p* = 0.46). This supports a model where shape comparisons are orientation sensitive, as one might expect, with rotation between sample and match reducing performance. However, we also found a significant interaction between the cross-modal contrast and rotation away from 0° on performance (b = 0.1, *t*_(27)_ = 4.14, *p* < 0.001). This suggests that orientation differences between sample and match have greater impact on performance for within-modal comparisons compared with cross-modal comparisons. Said another way, cross-modal recognition performance appears more invariant to rotation, a finding that accords with previous studies of visuohaptic recognition (Bülthoff and Edelman, 1992; Newell et al., 2001; Lacey et al., 2007, 2009; Andresen et al., 2009).

Finally, we analyzed the effect of rotation specifically within the unimodal VV and HH conditions. Looking at Figure 4, it appears that the effects of rotation were more pronounced for the within-modal haptic condition compared with the within-modal visual condition, and that the patterns of the effect were distinct. Using general linear hypothesis testing, we compared the 0–90°, 0–180°, and 90–180° rotation conditions within the VV and HH conditions. This analysis revealed that the effects of rotation for the visual matching trials were incremental and monotonic (0° vs 90°: b = 0.13, *z* = 2.149, *p* = 0.06; 90° vs 180°: b = 0.03, *z* = 0.59, *p* = 0.55; 0° vs 180°: b = 0.16, *z* = 2.74, *p* = 0.025). This suggests that for visual matching, performance degraded with increasing rotation. Rotation affected the haptic condition in a very different way. Rotation away from the original orientation by 90° dramatically affected performance (0° vs 90°: b = 0.35, *z* = 5.88, *p* < 0.0001), but a further 90° rotation actually improved performance (90° vs 180°: b = −0.16, *z* = −2.70, *p* = 0.025). We return to this distinction below, as we consider which specific shape dimensions appear critical for visual and haptic recognition and how these may differ.

Consistent with the match performance measures, each factor and their interaction significantly improved the model fit for reaction times (modality: χ^2^ (3) = 86.9, *p* < 0.001; rotation: χ^2^ (2) = 16.3, *p* = 0.0003; interaction: χ^2^ (6) = 38, *p* < 0.0001). These data are shown in Figure 4, and, as for the performance data, we can infer that modality, rotation, and their interaction are all significant predictors of reaction times.

Results of the contrast analyses revealed that reaction times are, as is evident from Figure 4, significantly faster for trials where the current stimulus is presented visually (visual current: b = −1068, *t*_(27)_ = 12.77, *p* < 0.0001). Reaction times for cross-modal trials were also significantly slower than unimodal trials (cross-modal: b = 241, *t*_(27)_ = 4.08, *p* < 0.0001). Rotation of a stimulus between sample and match also significantly slowed responses (b = 306, *t*_(27)_ = 5.12, *p* < 0.0001). Interactions between the modality and rotation factors also proved significant. The interaction between cross-modality and rotation was highly significant (b = −180, *t*_(27)_ = 3.01, *p* < 0.005), which corroborates the performance results presented above, again suggesting that the effects of rotation are less pronounced for cross-modal comparisons compared with within-modal comparisons. A significant interaction between the cross-modal contrast and the degree of rotation (90° vs 180°) conditions (b = −182, *t*_(72)_ = 2.62, *p* = 0.01) indicates that the 90° rotation slows responses for the within-modal condition but not the cross-modal condition. We also observed a significant interaction between the type of within-modal trial (visual/VV or haptic/HH) and any rotation (b = −620, *t*_(27)_ = 2.59, *p* = 0.012) as well as the degree of rotation (b = −571, *t*_(72)_ = 2.06, *p* < 0.05). We note that, although these interactions suggest that the visual and haptic within-modal comparisons are differentially affected by stimulus rotation, their interpretation is made difficult by the large difference in overall reaction time between the VV and HH conditions (see Discussion).

### Change in performance over time

The present study was designed to produce steady-state performance for analysis. To that end, we provided enough training and a large enough stimulus set so as to minimize any improvement in performance over the course of the experiment. Comparing the performance (*d*′) between the first 50 and last 50 trials for each condition and each participant confirmed little if any improvement during the experimental session. In the VV task, *d*′ went from 3.44 to 3.38 on average (paired *t* test, *p* = 0.79), in the HH task, *d*′ went from 1.49 to 1.59 (paired *t* test, *p* = 0.71). In the cross-modal condition, *d*′ went from 1.35 to 1.27 (paired *t* test, *p* = 0.64).

### Predicting behavior based on shape metrics

**Figure 5.**
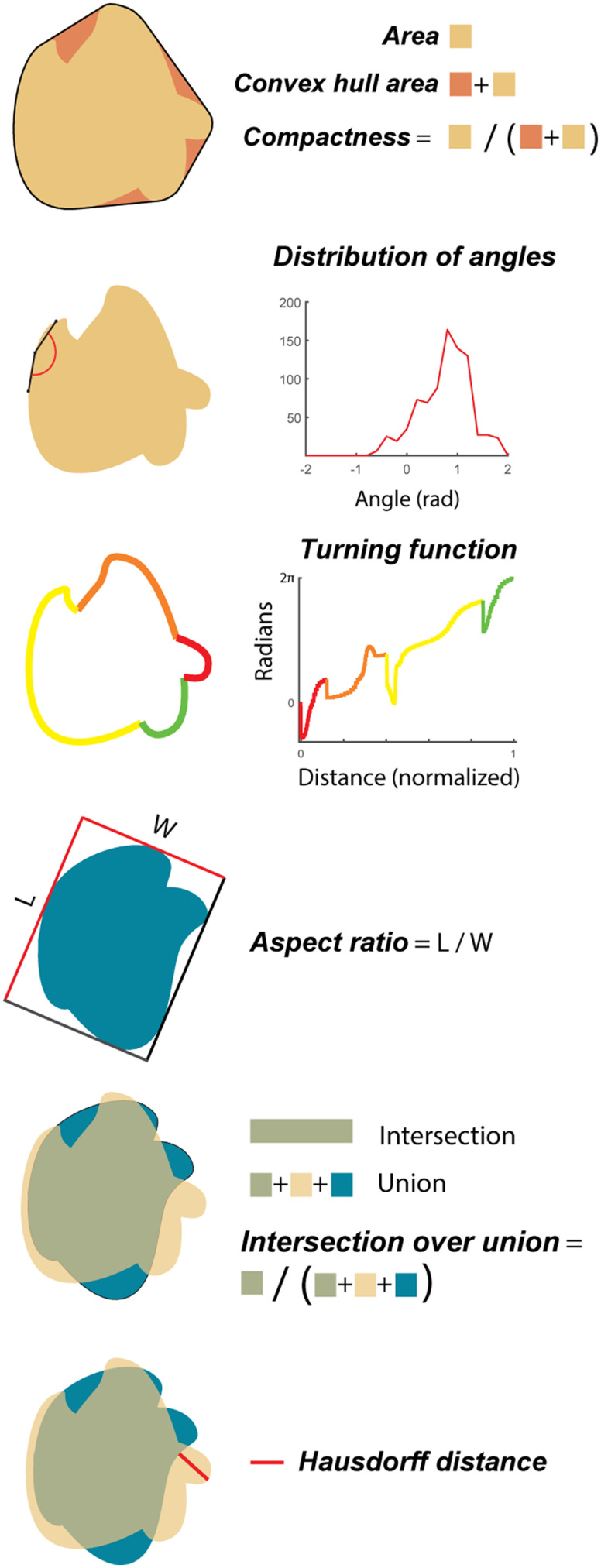
Shape comparison metrics. The metric “area” is the area encompassed by a shape. Convex hull area is the area encompassed by a boundary enclosing the shape with only convex turns. Compactness is the quotient of the area to the convex hull area. Distribution of angles represents all the angles that can be said to compose a given shape given a certain spread over which those angles are calculated. Turning function is a complete representation of a given shape using the tangent and length of each line segment composing that shape. This reformatting simplifies the comparison of shapes by being size and translation invariant. For explanatory purposes, an example shape is shown with color-coded sections. Aspect ratio (@optimal) is the largest ratio of length to width of a rectangle enclosing a shape. Intersection over union (@optimal) is the optimal overlap that can be achieved by overlaying one shape with another. The Hausdorff distance (@optimal) is the maximum of all minimum distances between all points on one shape and all points on another shape, optimized by rotating one shape relative to the other to find the smallest possible Hausdorff distance for a given pair of shapes.

The behavioral differences observed on the same task in different modalities led us to model the behavior to better understand the critical features of shapes participants used to complete this task and to compare these critical features for both visual and haptic shapes. To this end, we chose eight “shape metrics” (Fig. 5), which provide a variety of methods for quantifying the similarity between two shapes. Based on these shape metrics, we could then predict whether two shapes were likely to be conflated. By comparing the success of these various shape metrics in predicting behavior in each modality condition, we can gain insight into how shapes are evaluated by vision and touch. Details of each of these shape metrics are provided in Materials and Methods.

**Figure 6.**
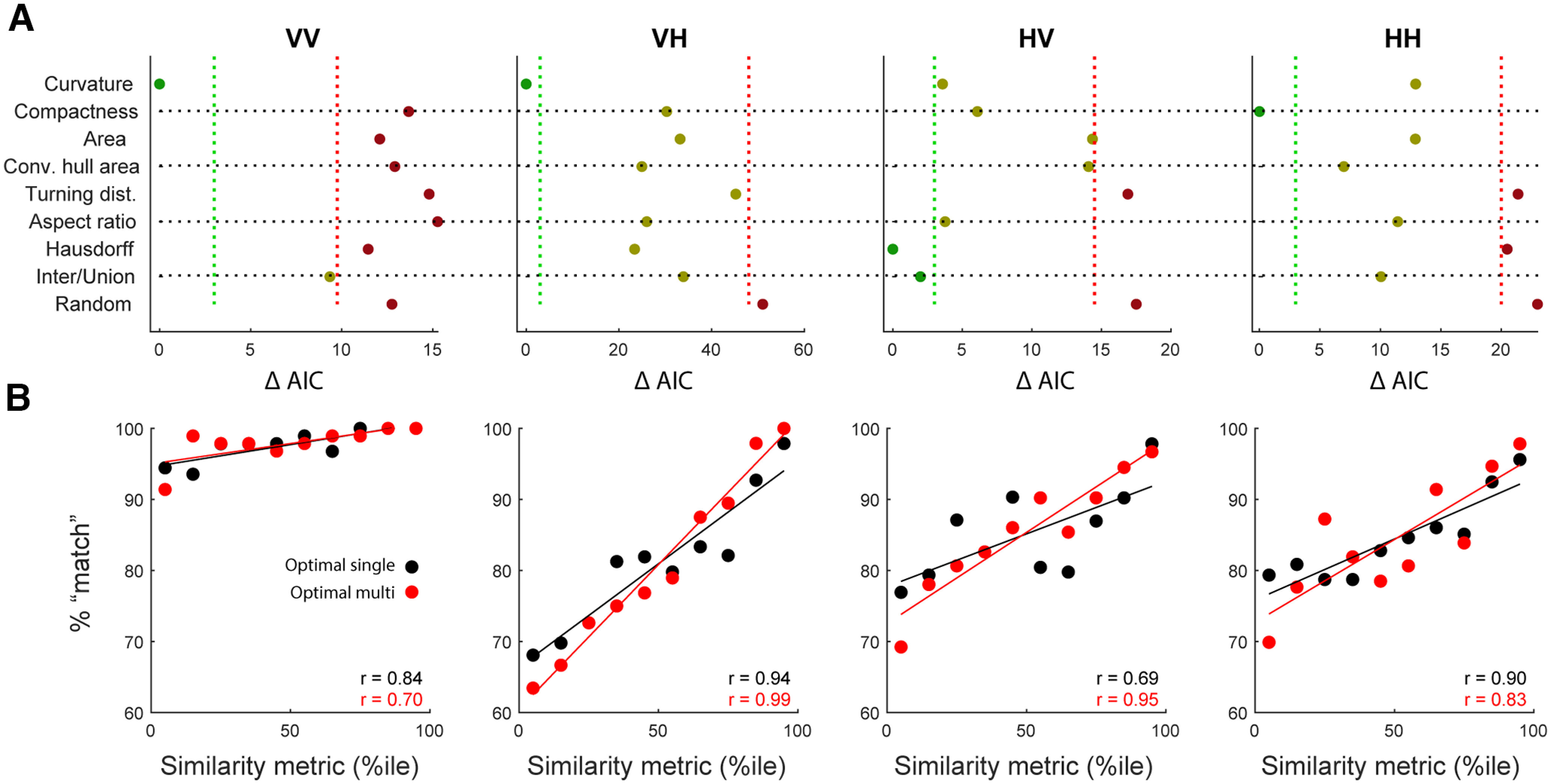
***A***, Performance for each similarity metric for each condition. The horizontal axis is the difference between the AIC of each individual metric and the best metric for that condition. Vertical dashed green lines indicate the threshold for what can be considered no different from the best model, while vertical dashed red lines indicate the boundary for metrics that can be considered no different from chance. ***B***, Behavioral performance on nonmatch trials correlates with the similarity of those shapes according to the most successful metric for that condition (optimal single, black). As the difference between two shapes increases, the probability that a participant will correctly label them as “nonmatch” increases. In all four conditions, the use of additional factors significantly improved predictive performance, although not necessarily leading to an increase in the correlation seen here. The optimal multimetric for VV and HH used two factors, while the optimal multimetric for HV and VH used three factors.

Figure 6 shows the performance of each model in fitting behavior for each of the four conditions. For within-modal visual comparisons, the single metric that best predicts behavior is the distribution of curvature. This metric simply catalogs the various angles that compose a given shape without regard for the spatial relationships between those angles. Similarly, for VH trials (comparing a haptic shape on the current trial with the visual shape seen on the previous trial), distribution of curvature is once again the best metric. For HV trials, the Hausdorff distance and intersection over union (@optimal) were most informative. The Hausdorff distance is a simple and extremely sparse description of the differences between shapes, representing the maximum of all minimum distances between points on a pair of shapes. If two shapes can be oriented such that their boundaries nearly overlap, the Hausdorff distance is small. However, if one of the two shapes has a large protrusion, but the shapes are otherwise identical, the Hausdorff distance is large. Intersection over union is the ratio of overlapping to nonoverlapping areas shared between two shapes. Finally, behavior on within-modal haptic trials is best described by the compactness metric. We can think of this metric as a measure of the area of the concavities of a shape (e.g., a circle is very compact because there are no concavities, whereas a starfish shape would not be compact). Considering the physical limitations inherent in the manual evaluation of an object, it is not so surprising that concavities are particularly salient in haptic exploration.

For a clearer sense of how well these metrics predict behavior, Figure 6 (bottom) shows the relationship between the shape difference (according to the best metric for that condition) and the performance of participants. It is important to note here that the best metric was not chosen according to which metric produces the strongest correlation, but rather according to which best predicts the choice of the participant on a trial-by-trial basis. Nevertheless, there is a clear, nearly monotonic, relationship in each condition in the expected direction: shapes that are more dissimilar according to a similarity metric are more likely to be labeled different by a participant (Pearson’s *r*; VV: *r* = 0.84, *p* = 0.002; VH: *r* = 0.94, *p* = 0.0001; HV: *r* = 0.69, *p* = 0.026; HH: *r* = 0.90, *p* = 0.0004). Although the trend is less pronounced in the VV condition, likely because of the near ceiling performance, the correlation is still highly significant. In all cases, performance for the most dissimilar shapes is nearly perfect. Figure 6*B* also shows the performance of the metrics when combined (red). Using AIC to “punish” models with added complexity, we found that the optimal models for each of the two within-modal conditions were best fit by combinations of two metrics, while each of the cross-modal conditions were best fit using combinations of three metrics. In all cases, these multimetrics were better able to predict behavior even after accounting for the added factors, though this does not necessarily result in a stronger correlation coefficient. For VV trials, the best multimetric was a combination of curvature and intersection over union, while for HH, it was compactness and hull area. Interestingly, for both VH and HV, the exact same three-factor multimetric was best: the combination of aspect ratio, curvature, and Hausdorff distance.

Three of the metrics described here were each calculated using two different methods, described here as @optimal and @actual. The intuition here is that we do not know *a priori* if behavior in this one-back matching task is better modeled by assuming participants are performing mental rotation (as they should, they were instructed to ignore rotation) or not. Ideally, participants would have a perfect recall of the shape presented on the previous trial and would have the ability to compare that shape with the shape presented on the current trial at all possible orientations and evaluate the similarity at each of those orientations. If there is any possible orientation where the shapes are a match, then the response is same; otherwise different. However, we know that mental rotation abilities are imperfect (Shepard and Metzler, 1971; Gauthier et al., 2002; Fig. 4), so it may be that performance is highly dependent on the exact orientation at which those shapes happen to be presented. If they happen to be oriented, for example, such that they both have a protrusion on top, they may be labeled same, while if those same shapes are presented with protrusions on opposite sides, they may be labeled different. Metrics that assess the similarity between two shapes at the optimal alignment that maximizes their similarity are labeled @optimal, while metrics that assess similarity at the actual orientations in which they were presented are labeled @actual.

**Figure 7.**
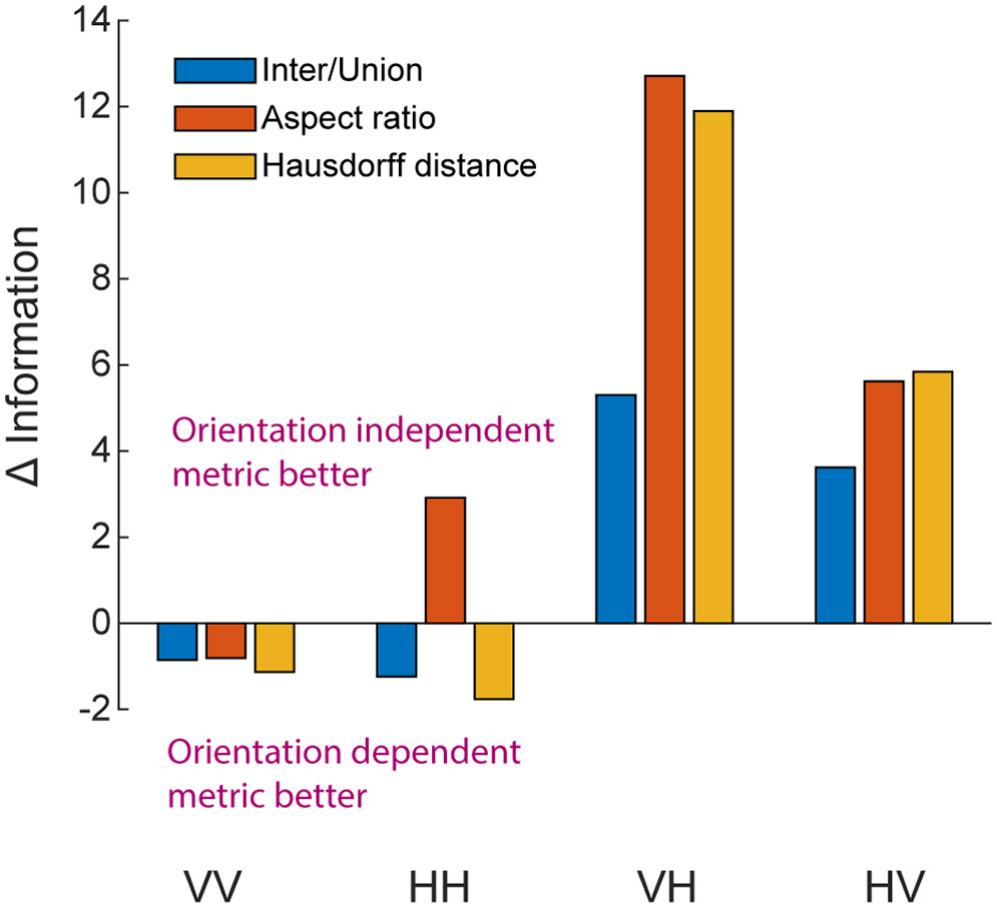
Within-modal versus cross-modal differences in behavior. Change in AIC between @optimal and @actual metrics show that within-modal behavior (conditions VV and HH) is better described by metrics that do not assume mental rotation (i.e., @actual metrics), while cross-modal behavior (conditions VH and HV) is better described by metrics that do assume mental rotation (i.e., @optimal metrics).

Interestingly, we found a clear difference between within-modal and cross-modal conditions in terms of whether they were better fit by @actual or @optimal metrics (Fig. 7). Within-modal behavior was better described by @actual metrics, and cross-modal behavior was better described by @optimal metrics. This implies that mental rotation is costly or difficult when comparing shapes within the same modality but simple or even automatic when comparing shapes across modality. This provides independent confirmation supporting the results presented above (Fig. 4) and reported previously (Lacey et al., 2009).

### Characterization of active exploration

**Figure 8.**
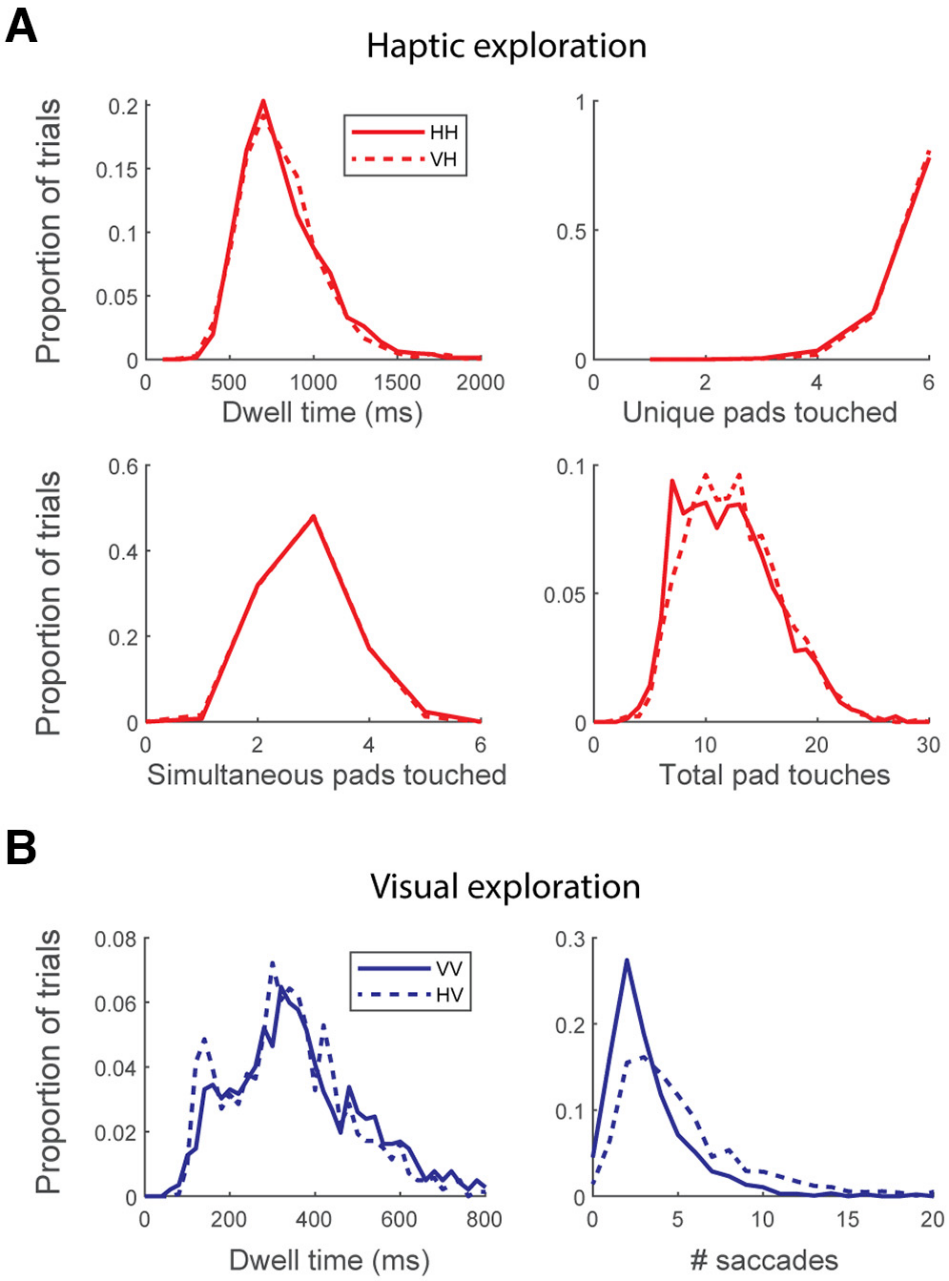
Statistics of haptic and visual exploration across all trials and participants. ***A***, Haptic exploration of shapes is remarkably consistent regardless of condition. Participants spent the same amount of time touching each portion of the shape (“dwell time”), touched the same proportion of the shape (“unique pads touched”), and touched the same proportion of the shape at once (“simultaneous pads touched”). The only difference is found in the duration of exploration, leading to more touches in the VH condition (total pad touches; K-S test, *p* = 0.009). ***B***, Similarly with visual exploration, the biggest difference between conditions is that participants spend more time looking around the shape in the HV condition (K-S test, *p* = 8e-47). Interestingly, the dwell time at each saccade end point is significantly shorter in the HV condition than in the VV condition (K-S test, *p* = 1e-5).

We also sought to determine whether the way participants touch shapes differs based on experimental condition (Fig. 8). Using the six touch sensors embedded in each shape, we quantified “dwell time” (the duration of each touch of a touchpad indicating how quickly the hand moves around the object), “unique pads touched” (how many of the six pads are touched on a given trial), “simultaneous pads touched” (how many pads are touched at any given moment, corresponding to the number of fingers being used), and “total pad touches” (the number of the six pads that were touched in the trial, an estimate of how much effort is spent on exploring a shape). No differences were found in dwell time, unique pads touched, or simultaneous pads touched between the HH and VH conditions, suggesting the basic strategy of how a shape is explored does not depend on the modality with which it is being compared. The only difference was found in the number of total pad touches, with more pad visits found in the VH condition [mean pad visits per trial: HH, 12.1; VH, 12.5; Kolmogorov–Smirnov (K-S) test, *p* = 0.009]. Interestingly, this difference in total pad touches between VH and HH conditions resulted almost entirely from the specific condition where the same haptic shape was presented consecutively (i.e., “match” trial) at the same orientation, corresponding to the relatively quick reaction times in this condition (Fig. 4, HH column, 0 change in angle). We conclude that people explore haptic objects the same whether comparing them to a previously presented haptic or visual shape, but that, as this comparison is more challenging, more time is spent carrying out that exploration.

To compare this with the visual behavior, we evaluated dwell time (i.e., intersaccade interval) and the number of saccades per trial. Similar to the results for haptic exploration, the biggest differences were seen in the amount of time exploring (mean saccades per trial: VV, 3.22; HV, 5.27; K-S test, *p* = 8e-47), again corresponding to differences in reaction time (Fig. 4). However, there was also a significant difference in the dwell time at each saccadic location, with participants making more frequent saccades in the HV condition (mean dwell time: VV, 405 ms; HV, 366 ms; K-S test, *p* = 1e-5).

**Figure 9.**
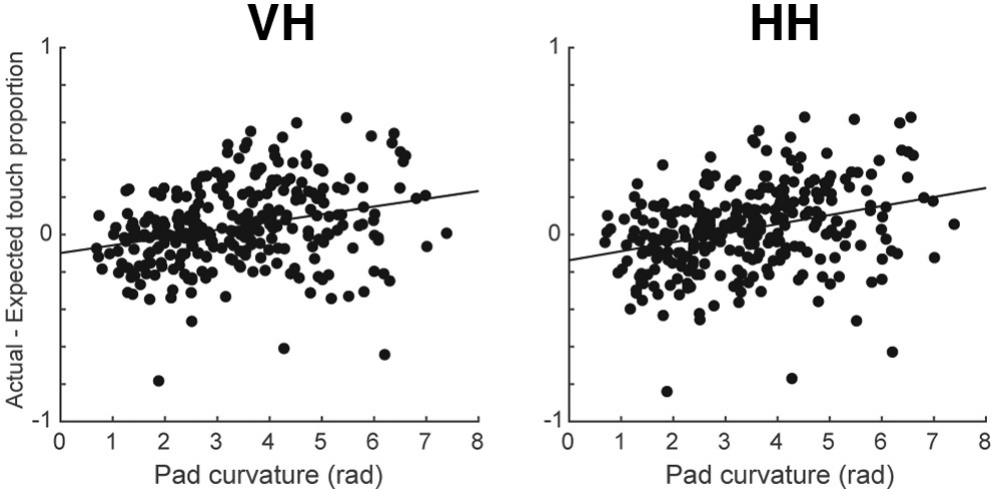
Regions with more curvature are touched more than expected based on Monte Carlo simulation. In both VH trials (left) and HH trials (right), touchpads with more curvature are touched more than would be expected by chance, whereas touchpads covering straighter portions of a shape are touched less than would be expected by chance. This indicates that participants specifically target areas of high curvature for manual exploration. Each point represents one touchpad on one shape. Line is the linear best fit. Pearson’s correlation. HH: *r* = −0.32, *p* = 4e-8; VH: *r* = 0.28, *p* = 1e-6.

Finally, we asked whether participants use targeted exploration to focus on areas of a shape with more curvature or whether exploration appears more uniformly distributed (Fig. 9). We used a Monte Carlo approach (details in Materials and Methods) to estimate the predicted touches of each touchpad if haptic exploration were random and then compared that to actual touches of those touchpads (we would not expect an equal number of touches for each of the six touchpads because not every pad is the same length or equally accessible to a finger). We found that, in both HH and VH conditions, the location of touches was not random, but instead there was a significant relationship between the length of time participants inspect a given touchpad and the amount of curvature in that area (Pearson’s correlation; HH: *r* = 0.32, *p* = 4e-8; VH: *r* = 0.28, *p* = 1e-6), suggesting that participants intentionally focus on exploring areas of high curvature that may contain more distinctive or “diagnostic” features.

## Discussion

In this study, human participants performed a one-back shape comparison task where they were presented with a continuous stream of shapes and asked to report whether the currently presented shape was the same as the previously presented shape. Shapes were presented either on a computer monitor (visual) or by a robotic arm to the participants’ left hand (haptic). Though our specific paradigm and the manner of presentation of physical objects were novel, we did confirm previous results found in visual–haptic research (Bülthoff and Edelman, 1992; Newell et al., 2001; Lacey et al., 2007, 2009; Andresen et al., 2009), showing that orientation is important when comparing shapes within a modality but not when comparing shapes across modalities. This increases our confidence that the results shown here are broadly applicable for visual–haptic research using different stimuli and different presentation methods.

The primary new findings in the present study are that (1) performance in this shape-matching task is predictable based on simple metrics that quantify the dissimilarity between shapes, and (2) that the metrics that best predict behavior depend on the presentation modality. For example, the best metric for predicting a within-modal visual comparison is very different from the best metric for predicting behavior on a within-modal haptic comparison. This does not appear to result from simple differences in spatial acuity between the senses but rather from fundamental differences in the way shapes are processed.

These results may seem to contradict a recent study by Tabrik et al. (2021). In that study, a great deal of similarity was found between the chosen shape metrics and the self-reported measures of perceptual similarity in both within-modal visual and within-modal haptic comparisons (different groups of participants were used for these tasks, so no cross-modal comparisons were possible). A number of differences between that study and the present study could explain the discrepancy. First, it may be that self-report of perceived shape similarity on a 7-point scale is different from the “revealed perceptual similarity” obtained in the present study, where we analyze which shapes are confused for other shapes. For example, a participant may assess that two shapes are very similar to each other overall and yet the small difference could be quite salient such that they would never be confused for each other. Second, the Tabrik et al. (2021) study used shapes that were generated by evolving eight shapes from each of two related initial shapes using digital embryo algorithms. Because the shapes were all part of the same family, that may have encouraged the evaluation of shape differences on a given set of dimensions that best describe the differences in that specific shape family but would not necessarily describe differences in independently generated shapes. Third, the Tabrik et al. (2021) study allowed participants to manipulate the shapes (physically in the haptic condition, virtually in the visual condition), whereas the present study did not. This raises the interesting possibility that active manipulation of a shape may alter the perceived similarity between shapes.

One difference between the within-modal and cross-modal tasks used here is that of task switching. That is, in VV and HH conditions, each trial is in the same modality as the previous one and thereby requires the same physical and cognitive processes. The cross-modal task demands a constant switching between modalities from trial to trial. It is possible that this task switching leads to a significant increase in cognitive load and thereby impairs performance compared with what would otherwise be expected. Using a same–different task structure where each block is only VH or HV comparisons rather than the one-back task used here may lead to improved performance. Although there is still a constant switching of modality required, there is more consistency in that the participant is repeatedly asked to compare the current visual stimulus to a remembered haptic stimulus and vice versa in separate blocks. The lack of difference in performance (Fig. 3) between cross-modal and HH trials suggests that any increase in task‐switching demand is minimal, and we believe the differences between VV and HV performance is better explained by the VV condition simply being fundamentally easier in this task, which also explains why VV performance is much better than HH, neither of which require task switching.

The shapes used in the present study were two-dimensional for visual presentation and extruded two-dimensional shapes for haptic presentation. As opposed to more complex three-dimensional stimuli, this allows for greater control of the available stimulus information to the participant and more inherent similarity between the two modalities (e.g., the back of an object is not visible but can be touched). Although we are unaware of any reason to assume that more complex three-dimensional stimuli would yield a different result, we also cannot rule that out.

It is important to note that the metrics used here to quantify differences between shapes can be used predictively. That is, it should be possible to intentionally create shape sets that are difficult to differentiate visually or haptically. Furthermore, it should be possible to create stimulus sets that are difficult to differentiate haptically but easy to differentiate visually, and vice versa. Some previous work in this area has used *post hoc* analyses of behavior to group shapes by similarity, but did not provide a means of directly predicting perceived similarity in the absence of behavioral results (Huang, 2020). We sought here to develop models that are more readily interpretable and can thereby provide greater intuition (Rudin et al., 2021).

It is also important to emphasize that object familiarity may play a role in the extent to which different brain areas are involved in visual and haptic object recognition. Previous work (Deshpande et al., 2010; Lacey et al., 2010) has indicated that the networks involved in haptic object recognition are similar to visual object recognition only when the shapes are familiar. This may also explain the results found in the study by Tabrik et al. (2021) where shapes were likely more familiar and greater similarity was found between visual and haptic processing compared with the present study. Further work will seek to determine the extent to which stimulus familiarity impacts which metrics best to predict human behavior.

Finally, this work suggests something about shape processing in the brain, more generally. Our initial hypothesis was that, to the extent that haptic object recognition recruits visual cortical areas for processing object shape, the same properties that are important for differentiating visual shapes should be important for differentiating haptic shapes. For example, visual shape recognition is thought to rely on combining the activity of neurons in visual cortex sensitive to local curvature (Riesenhuber and Poggio, 1999; Serre et al., 2005; Yau et al., 2009; Pasupathy et al., 2020). This would predict that two different shapes with similar local curvature would be easily confused. If haptic shape recognition uses the same pathways, we would expect local curvature similarities to also contribute to mistakes of haptic shape recognition, particularly if we allow for different definitions of “local” based on the lower acuity of haptic versus visual perceptions (i.e., measuring local curvature over various distances that should be optimized based on fingertip size). This does not appear to be the case. Rather, the metrics that work well for predicting haptic–haptic shape comparison appear fundamentally different from those that work well for predicting visual–visual shape comparison. This suggests that pathways for within-modal haptic shape processing may exist somewhat independent of visual processing. While these pathways have not yet been fully discovered, the device developed in this study for presenting haptic objects could be used to explore these circuits more systematically than has been possible in previous studies.

The finding that behavior in the cross-modal conditions is best fit by models that combine three metrics, while behavior in the within-modal conditions is best fit by models that combine only two metrics, further bolsters the view that processing across modalities is fundamentally different from processing within modality. The observed increase in complexity required to explain behavior may reflect an increase in complexity of the networks involved and the need for interactions between these, which can perhaps be short-circuited for within-modal comparisons, most notably in the absence of rotation. Further work, particularly using electrophysiology and neuroimaging techniques, should prove useful in elucidating the areas that are involved in these varying shape recognition scenarios.

## Synthesis

Reviewing Editor: David Schoppik, New York University - Langone Medical Center Decisions are customarily a result of the Reviewing Editor and the peer reviewers coming together and discussing their recommendations until a consensus is reached. When revisions are invited, a fact-based synthesis statement explaining their decision and outlining what is needed to prepare a revision will be listed below. The following reviewer(s) agreed to reveal their identity: Thomas James, Bevil Conway.

We felt that the major advance of the manuscript was methodological. For example, “It presents a creative and interesting paradigm that looks like it could open up a new field of research” and “The studies are very carefully designed and the experiments were carried out to the highest standards.” In contrast, though, the reviewers felt that “The more theoretically-driven / experimental-testing part of the manuscript is quite lacking,” commenting on the conclusions being much too strong, and inadequate consdieration of the literature. Consequentially, the manuscript is best suited to be reworked as a submission in the “Tools” category rather than a full-on research piece. That would allow the strengths of the manuscript to shine. Below, I detail the most salient of the specific critiques from the review / discussion. As a “Tools” paper these would not all need to be addressed, but it was our sincere hope that these might be beneficial no matter how you choose to proceed.

1) This quote from the abstract and similar statements throughout “One might expect that the processes of visual and haptic object recognition engage similar brain structures given the similarity of the problem and the advantages of avoiding redundant brain circuitry, but this has not yet been established” demonstrates a lack of knowledge of the neuroimaging literature on visuo-haptic object recognition. In fact, there is quite a bit of evidence for shared computations in the brain for visual and haptic object recognition. Many of these contributions are cited in Snow et al. 2015, which the authors cite, so it is surprising that they don’t acknowledge them. In particular, James et al. 2002 used a cross-modal priming task, which is relevant to the current study in that it relies on memory (loosely) to assess interactions between vision and haptics. Also, of interest is James et al. 2005, which documents a haptic object recognition deficit in patient DF that is as severe as her visual object recognition deficit. Further, it would seem to me one element is missing: what we use haptic and visual shape information to do would well be different, and presumably would necessitate different neural mechanisms (and be evident in differences in behavioral performance).

2) There is a literature on “areal” vs “neuronal” convergence that seems somewhat related to the “early” vs “late” distinction made here. However, to assess these ideas requires some type of “summation” task. In multisensory studies, this is usually accomplished with each sensory stimulus presented alone and compared to them presented combined. The task used here is ok, but I’m not sure that I would make such strong claims about brain machinery having assessed behavior with only a single task and especially a task that does not require combination of the sensory inputs. Papers by Kim and James around 2010 made use of this summation principle to assess visuohaptic convergence using fMRI and, again, found evidence for shared machinery (which is also missing from the lit review).

3) The use of a one-back design is very efficient, but I think it poses a problem for directly comparing uni-sensory and multi-sensory recognition. In the unimodal runs/blocks, the encoding and retrieval modalities are constant. However, in the multimodal blocks, the encoding and retrieval modalities switch on every trial. The task-switching costs in this scenario could be severe and may lead to the differences in performance. Behavioral priming studies from the 1980s (Greene & Srinivan; Belesteros) showed similar priming effects for within-modal and between-modal conditions, suggesting shared brain machinery (or cognitive representation). Newer work by Kim and James (mentioned above) found a very small effect of combining vision and haptics. The effects reported here are strikingly large given the lack of previous evidence for such differences and therefore may be more attributable to task-switching difficulty than a difficulty combining information across modalities. It may have been better in this case to use isolated trials, rather than a continuous stream as done in the one-back.

4) The stimulus delivery method was different for vision and haptics. Thus, for within modal conditions, the delivery method was the same for both vision-vision and haptics-haptic. But, for the between-modal conditions, the delivery method from encoding to retrieval was different. This puts the between-modal conditions at a performance disadvantage compared to the within-modal conditions and could help explain the very large effects.

Further, it seems pretty clear from the results, e.g. in Figure 3, that the discrimination is simply worse for any trials that use the haptic condition compared to the visual condition. The analysis in lines 243 to 252 advance an interpretation of order effects, but what data support this? Figure 3a shows that the percent correct of HV and VH are “n.s.”, and Figure 3b shows that the d’ is similarly insignificant for comparing HV and VH. It seems to me that it’s just the visual stimuli are more accurately perceived, and this masquerades as an order effect because of something funky in the way the analysis is conducted. Moreover, there doesn’t seem to be any worse performance for cross modal compared to just the haptic-haptic, suggesting that the poorer performance on the cross-modal tasks is simply because of the haptic performance. The results present analyses that suggest that there were differences in cross-modal versus within modal (line 250). I’m not sure on what this is based. Certainly the VV is different from the HV and VH; but the HH isn’t different from the HV and VH. A little further on, the paper claims that the cross-modal reaction times were longer than the within-modal reaction times (lines 260), but this comparison is “n.s” in Figure 3 (and doesn’t look significant to me).

Line 263. I have no fetish for a p-value threshold of 0.05, but I think it’s risky to conclude “marginal significance” with a p value of 0.097 (which is really 0.1, 10%, not so far from the belly of the normal distribution).

Figure 4, the cross-modality reduces performance even with no rotation, suggesting that the results are not a rotation-specific-to-within-modality effect.

5) The apparatus presents 2D “blobs”. Personally, I like the “blobs” idea and there is certainly a lot that can be learned from studying 2D stimuli. However, the conclusions that can be made about the integration of visual and haptic object recognition is limited by the use of only 2D stimuli here. A full accounting would require tasks that also used 3D stimuli. Thus, I feel that the conclusions are too strong, given the limited scope of the stimuli.

6) A caution when interpreting behavioral data with respect to brain mechanisms is that one must keep in mind that behavior is a reflection of the whole brain, not one part of it. As described above, there is a decent literature implicating the lateral occipital cortex and anterior intraparietal sulcus in the combination of visual and haptic shape. But, the behavioral task used here does not just measure the function of those two regions. In the extreme, the question of whether vision and haptics use the same machinery is hardly necessary to ask, because vision uses the eyes and haptics uses the skin, tendons, etc., so obviously they are different. In other words, it is not so surprising/interesting to find that vision and haptics are different, but it is surprising/interesting when they are found to be the same.

7) The number of participants seems light. We all acknowledge that we are still in the Time of Pandemic, so maybe this is why. But there is quite a lot of variability in the performance of the ten participants, which suggests that a sample of a larger number of people would be needed to come to any firm conclusions. The paper describes many trends; another clue that more data might be called for.

8) Related to the point above, it seems like the tasks in the paper are the sorts of tasks that people would get better at, and I wondered if the authors have any data/comment on the learning, and/or whether the learning rates differ substantially for the various conditions (VV, HV, VH, HH).

## Author Response

We felt that the major advance of the manuscript was methodological. For example, “It presents a creative and interesting paradigm that looks like it could open up a new field of research” and “The studies are very carefully designed and the experiments were carried out to the highest standards.” In contrast, though, the reviewers felt that “The more theoretically-driven / experimental-testing part of the manuscript is quite lacking,” commenting on the conclusions being much too strong, and inadequate consideration of relevant literature. Consequentially, the manuscript is best suited to be reworked as a submission in the “Tools” category rather than a full-on research piece. That would allow the strengths of the manuscript to shine. Below, I detail the most salient of the specific critiques from the review / discussion. As a “Tools” paper these would not all need to be addressed, but it was our sincere hope that these might be beneficial no matter how you choose to proceed.

We thank the reviewers for the suggestion and agree that a submission in the "Tools” category would be a great fit.

1) This quote from the abstract and similar statements throughout “One might expect that the processes of visual and haptic object recognition engage similar brain structures given the similarity of the problem and the advantages of avoiding redundant brain circuitry, but this has not yet been established” demonstrates a lack of knowledge of the neuroimaging literature on visuo-haptic object recognition. In fact, there is quite a bit of evidence for shared computations in the brain for visual and haptic object recognition. Many of these contributions are cited in Snow et al. 2015, which the authors cite, so it is surprising that they don’t acknowledge them. In particular, James et al. 2002 used a cross-modal priming task, which is relevant to the current study in that it relies on memory (loosely) to assess interactions between vision and haptics. Also, of interest is James et al. 2005, which documents a haptic object recognition deficit in patient DF that is as severe as her visual object recognition deficit. Further, it would seem to me one element is missing: what we use haptic and visual shape information to do would well be different, and presumably would necessitate different neural mechanisms (and be evident in differences in behavioral performance).

Reviewers are correct to point out that there is a fairly extensive literature suggesting the processing of visual and haptic shapes in overlapping brain areas, and indeed we did cite many of these papers. Our belief, however, is that the question of the exact nature of the computational role of this potential overlap remains unresolved. In particular, we think much of the activation of ventral cortical areas (primarily “tactile LOC”) may be rooted in mental imagery and/or visual input due to the participants’ eyes being open and making numerous saccades during palpation. Evidence for the former comes largely from the relative lack of LOC activation with novel haptic stimuli (James 2002, Cacciamani & Likova, 2016; Deshpande et al., 2010; Lacey et al., 2010), while evidence for the latter comes from our own neural recordings from monkeys during a haptic task where ventral cortical neurons seem to be robustly driven by eye movements with no visual stimuli on a screen. The current paper doesn’t provide conclusive resolution to this question, so we suggested (rather ineloquently) that the question “has yet to be established” or similar. In hindsight, we agree this topic deserves clarification and elaboration. We did try to address this in the discussion, but we have also now made changes throughout the abstract, significance statement, and introduction to address this issue.

2) There is a literature on “areal” vs “neuronal” convergence that seems somewhat related to the “early” vs “late” distinction made here. However, to assess these ideas requires some type of “summation” task. In multisensory studies, this is usually accomplished with each sensory stimulus presented alone and compared to them presented combined. The task used here is ok, but I’m not sure that I would make such strong claims about brain machinery having assessed behavior with only a single task and especially a task that does not require combination of the sensory inputs. Papers by Kim and James around 2010 made use of this summation principle to assess visuohaptic convergence using fMRI and, again, found evidence for shared machinery (which is also missing from the lit review).

Agreed. In this task we are focusing on unisensory rather than multisensory representations so as to simplify interpretation.

3) The use of a one-back design is very efficient, but I think it poses a problem for directly comparing uni-sensory and multi-sensory recognition. In the unimodal runs/blocks, the encoding and retrieval modalities are constant. However, in the multimodal blocks, the encoding and retrieval modalities switch on every trial. The task-switching costs in this scenario could be severe and may lead to the differences in performance. Behavioral priming studies from the 1980s (Greene & Srinivan; Belesteros) showed similar priming effects for within-modal and between-modal conditions, suggesting shared brain machinery (or cognitive representation). Newer work by Kim and James (mentioned above) found a very small effect of combining vision and haptics. The effects reported here are strikingly large given the lack of previous evidence for such differences and therefore may be more attributable to task-switching difficulty than a difficulty combining information across modalities. It may have been better in this case to use isolated trials, rather than a continuous stream as done in the one-back.

This is an interesting and important point. It’s not clear to us that there are considerable switching cost differences considering the task (cognitively speaking) is always the same, but it is plausible and we agree it should be noted and have addressed this in the discussion. That said, we’re not quite sure what the reviewer is referring to when describing the results as “strikingly large”. Reaction times for within-modal trials were only modestly higher than cross-modal (p=0.029) and were not significantly faster in either VV vs. HV or HH vs. VH. Similarly, neither cross-modal condition led to more mistakes than the HH condition, although the VV condition was comparably easy and drives the finding that within-modal performance is better than cross-modal. These results suggest to us that any task-switching cost is minimal.

4) The stimulus delivery method was different for vision and haptics. Thus, for within modal conditions, the delivery method was the same for both vision-vision and haptics-haptic. But, for the between-modal conditions, the delivery method from encoding to retrieval was different. This puts the between-modal conditions at a performance disadvantage compared to the within-modal conditions and could help explain the very large effects. Following the thread from the previous question, as we would characterize the cross-modal impact as small, we take this as evidence that the different delivery methods for the two modalities does not play a large role. Further, it seems pretty clear from the results, e.g. in Figure 3, that the discrimination is simply worse for any trials that use the haptic condition compared to the visual condition. The analysis in lines 243 to 252 advance an interpretation of order effects, but what data support this?

Figure 3a shows that the percent correct of HV and VH are “n.s.”, and Figure 3b shows that the d’ is similarly insignificant for comparing HV and VH. It seems to me that it’s just the visual stimuli are more accurately perceived, and this masquerades as an order effect because of something funky in the way the analysis is conducted. Moreover, there doesn’t seem to be any worse performance for cross modal compared to just the haptic-haptic, suggesting that the poorer performance on the cross-modal tasks is simply because of the haptic performance. The results present analyses that suggest that there were differences in cross-modal versus within modal (line 250). I’m not sure on what this is based. Certainly the VV is different from the HV and VH; but the HH isn’t different from the HV and VH. A little further on, the paper claims that the cross-modal reaction times were longer than the within-modal reaction times (lines 260), but this comparison is “n.s” in Figure 3 (and doesn’t look significant to me).

Thank you for pointing this out. The analysis was showing that (VV,VH) > (HV,HH) and (VV,HV) > (VH,HH) but we see how it implied an argument for order effects that is really more confusing than illuminating considering how they are obviously just driven by the high VV performance. We’ve removed the three related sentences. We’ve also added a clause to emphasize that the within-modal performance is better than cross-modal primarily due to the VV condition. Line 263. I have no fetish for a p-value threshold of 0.05, but I think it’s risky to conclude "marginal significance” with a p value of 0.097 (which is really 0.1, 10%, not so far from the belly of the normal distribution).

Agreed, this was not the correct description for p=0.097. Changed to “not significant”. Figure 4, the cross-modality reduces performance even with no rotation, suggesting that the results are not a rotation-specific-to-within-modality effect. Comparing VH and HH, it may be more accurate to frame it as “within-modality improves performance with no rotation”. Cross-modal performance tends to be more in line with within-modal rotated performance whereas trials with no change in either modality or angle seem to be a special case.

5) The apparatus presents 2D “blobs”. Personally, I like the “blobs” idea and there is certainly a lot that can be learned from studying 2D stimuli. However, the conclusions that can be made about the integration of visual and haptic object recognition is limited by the use of only 2D stimuli here. A full accounting would require tasks that also used 3D stimuli. Thus, I feel that the conclusions are too strong, given the limited scope of the stimuli. This is an important point and we’ve added a section to the discussion to acknowledge this. We elected to explore these 2D (and 2D extruded) shapes because they can be compared across modality in a straightforward way.

6) A caution when interpreting behavioral data with respect to brain mechanisms is that one must keep in mind that behavior is a reflection of the whole brain, not one part of it. As described above, there is a decent literature implicating the lateral occipital cortex and anterior intraparietal sulcus in the combination of visual and haptic shape. But, the behavioral task used here does not just measure the function of those two regions. In the extreme, the question of whether vision and haptics use the same machinery is hardly necessary to ask, because vision uses the eyes and haptics uses the skin, tendons, etc., so obviously they are different. In other words, it is not so surprising/interesting to find that vision and haptics are different, but it is surprising/interesting when they are found to be the same. Agreed, although we would say that it becomes more interesting as a contrast to papers finding them to be the same!

7) The number of participants seems light. We all acknowledge that we are still in the Time of Pandemic, so maybe this is why. But there is quite a lot of variability in the performance of the ten participants, which suggests that a sample of a larger number of people would be needed to come to any firm conclusions. The paper describes many trends; another clue that more data might be called for. Indeed, the pandemic certainly played a role and in response to this and other points we have rephrased many of our summary statements.

8) Related to the point above, it seems like the tasks in the paper are the sorts of tasks that people would get better at, and I wondered if the authors have any data/comment on the learning, and/or whether the learning rates differ substantially for the various conditions (VV, HV, VH, HH).

This is a good question. We did explore changes in performance over time and found very little change over the course of the experiment. Slight (insignificant) improvement for HH and slight (insignificant) worsening for VV and VH/HV. Because there were no clear trends, we left it out but it warrants mentioning that we did explore this, which we now have in a paragraph in the results section. It’s certainly plausible that more extensive training would have yielded more conclusive effects of experience.
